# Antitumor effects of two bisdioxopiperazines against two experimental lung cancer models *in vivo*

**DOI:** 10.1186/1471-2210-4-32

**Published:** 2004-12-24

**Authors:** Da-Yong Lu, Bin Xu, Jian Ding

**Affiliations:** 1 Division of Anticancer Pharmacology, Shanghai Institute of Materia Medica, Shanghai Institutes of Biology Sciences, Chinese Academy of Sciences, Shanghai 201203, PR China; 2 School of Life Sciences, Shanghai University, Shanghai 200436, PR China; 3 Graduate School of Chinese Academy of Sciences, PR China

## Abstract

**Background:**

Probimane (Pro), an anti-cancer agent originating in China, was derived from razoxane (ICRF-159, Raz), a drug created in Britain, specifically targeting at cancer metastasis and as a cardioprotectant of anthrocyclines. Pro and Raz are bisdioxopiperazine compounds. In this work, we evaluated the anti-tumor and anti-metastatic effects of Pro and Raz *in vivo *against two lung tumor models, one of murine origin (Lewis lung carcinoma, LLC) and one of human origin (LAX-83).

**Results:**

After determining the lethal dosage of Pro and Raz, we assessed and compared the inhibitory effects of Pro and Raz against primary tumor growth and metastatic occurrences of LLC at the dosage of LD_5_. Pro and Raz were active against primary tumor growth and significantly inhibited pulmonary metastasis of LLC at same dose-ranges (inhibitory rates > 90 %). Both Raz and Pro were effective in 1, 5, and 9 day administration schedules. Three different schedules of Raz and Pro were effective against the primary tumor growth of LLC (35–50 %). The synergistic anticancer effect of Raz with bleomycin (Ble) (from 41.3 % to 73.3 %) was more obvious than those with daunorubicin (Dau) (from 33.1 % to 56.3 %) in the LLC tumor model. Pro was also seen to have synergistic anti-cancer effects with Ble in the LLC model. Both Raz and Pro inhibited the growth of LAX 83 in a statistically significant manner.

**Conclusions:**

These data suggest that both Raz and Pro may have anti-tumor potentiality and Raz and Pro have combinative effects with Ble or Dau. The potential targets of bisdioxopiperazines may include lung cancers, especially on tumor metastasis. The anti-cancer effects of Raz and Pro can be increased with the help of other anticancer drugs.

## Background

Razoxane (ICRF-159) (*Raz*), first developed in UK, was the earliest agent against spontaneous metastasis for the murine model (Lewis lung carcinoma) in 1969 [1]. A large volume of papers and projects have been published in the utilities and mechanisms of *Raz *for anticancer actions, like assisting radiotherapy, [2] overcoming multi-drug resistance (MDR) of daunorubicin and doxorubicin [3], inhibiting topoisomerase II [4] and so on. More importantly, *Raz*, as a cardioprotectant of anthrocyclines, has been licensed in 28 countries in 4 continents. Since morpholine groups in some structures were reported to be responsible for cytotoxic or modulative actions on tumors, an anticancer agent, probimane [1,2-bis (N^4^-morpholine-3, 5-dioxopeprazine-1-yl) propane; AT-2153, Pro] was synthesized by introducing two morpholine groups into *Raz *in China.[5]. *Raz *and *Pro *belong to *bisdiopiperazines*. Like *Raz*, *Pro *also exhibits anti-tumor activity both *in vivo *and *in vitro *against experimental tumor models in a small scale investigation [6,7] and limited clinical data showed that *Pro *could inhibit human malignant lymphoma even for those resistant to other anticancer drugs [8]. Pro exhibits the same pharmacological effects as *Raz*, like detoxication of *Adriamycin *(*ADR*) induced cardiotoxicities, and synergism with *ADR *against tumors [9,10]. We have found some novel biological effects of *Pro*, like inhibiting the activity of calmodulin (*CaM*), a cell-signal regulator, which can explain anticancer actions and the combined cytotoxic effect of *Pro *and *ADR *[11]. Pro was also shown to inhibit lipoperoxidation (*LPO*) of erythrocytes [12], influence tumor sialic acid synthesis [13] and inhibit the binding of fibrinogen to leukemia cells [14].

Lung cancer is the No 1 killer among all categories of cancers in urban areas in China and many Western countries. The high mortality rate of lung cancer can easily be caused by inducing multi-drug resistance (*MDR*) and by high metastatic occurrence in clinics [15]. Since we assume that *Pro*, like *Raz *may possess useful therapeutic potentialities, we evaluated *in vivo *the chemotherapeutical parameters of *Pro *and *Raz *for lung cancer of both murine and human origins.

## Results

### Lethal toxicity of Pro and Raz in mice

The lethal dosage of *Pro *and *Raz *is tabulated in Table 1. Since the toxicity of *Pro *and *Raz *seemed to lack sex specificity in mice, we were able to combine their numbers for LD_50 _and LD_5 _calculations. We used the approximate dosage of LD_5 _of Pro (60 mg/kg ip × 7) and Raz (20 mg/kg ip × 7) as equitoxic dosages for further treatment studies.

**Table 1:** The subacute toxicity of Pro and Raz in mice: Mouse survival was observed for 1 month. The numbers of mice in each group were 20 for each of the 5 dosages of a single agent.

Drugs	Protocols	LD_5 _mg/kg	LD_50 _mg/kg
Probimane	ip × 10	66	121
Razoxane	ip × 10	23	53

### Antitumor and antimetastatic effects of Pro and Raz on LLC

Antitumor and antimetastatic effects of *Pro *and *Raz *on *LLC *are tabulated in Table 2 and Table 3. *Pro *and *Raz *at equitoxic dosages (LD_5_) showed a noticeable anticancer effect on primary tumor growth (inhibitory rates, approximately 30–45 %), and significantly inhibited the formation of tumor metastases (inhibitory rates on pulmonary metastasis > 90 %, P < 0.001). Primary tumor growth of *LLC *was inhibited more by *Pro *(48 %) than by *Raz *(40.3%) in a 20 day trial, whereas the inhibition of *Pro *(35.7%) was slightly less than that of *Raz *(40 %) on an 11 day trial. Pro seems to be more persistent than Raz in inhibiting primary tumor growth of *LLC*.

### Antitumor effects of bisdioxopiperazines for different schedules and in combination with other anticancer drugs

Antitumor effects of *Raz *and *Pro *on *LLC *are included in Table 4, 5, 6. We evaluated 1, 5 and 9 day administration schedules in our study. We found that *Raz *and *Pro *were effective in a statistically significant manner with the 3 injection schedule of the 1, 5 and 9 day administrations on *LLC*. If we administered *Raz *to tumor-bearing mice once on day 1, 5 and 9, there was no difference between treatment and vehicle control. Antitumor effects of *Raz *in combination with *Ble *on *LLC *(73.3 %) were better than those in combination with *Dau *(56.3 %) (Table 5 and Table 6). *Pro *also showed synergistic effects in combination with *Ble *(Table 7).

**Table 2:** The influence of Pro and Raz on primary tumor of LLC (using Student T-test): Route: ip × 7 daily. Experiment term was 11 days. * P < 0.05 (treatment vs vehicle control). The numbers of mice were 30 for the control group and 20 for each treatment group. 100 % survival was observed in each group.

Compounds	Dosage mg/kg/d	Body weight (g)	Tumor weight (g)	Tumor inhibition%
Control	--	23.3/24.4	2.80 ± 0.04	--
Razoxane	20	23.3/23.4	1.61 ± 0.03*	40.0
Probimane	30	23.4/21.6	1.91 ± 0.03*	32.1
Probimane	60	23.3/23.8	1.80 ± 0.03*	35.7

**Table 3:** The influence of Pro and Raz on primary and metastatic tumor of LLC: PTI (%) – Primary tumor inhibition. MFCPM – metastatic foci count per mouse. Route: ip × 7 every 2 days. Experiment term was 20 days, * P < 0.001(treatment vs vehicle control). The numbers of mice were 30 for both control group and each treatment group. 100 % survival was observed in each group.

Compounds	Dosage mg/kg/d	Body weigh (g)	PTI(%)	MFCPM
Control	---	22.8/21.4	--	30.9 ± 7.3
Razoxane	20	22.7/21.5	40.3	1.2 ± 0.5*
Probimane	30	23.3/22.5	42.0	1.5 ± 0.5*
Probimane	60	23.3/20.3	48.0	1.0 ± 0.2*

**Table 4:** Antitumor effects of bisdioxopiperazines of different schedules on Lewis lung carcinoma: *Administration every 3 hours, 16 mice were included in each testing group. **p < 0.05 (treatment vs control), Experimental term was 11 days

Compounds	Dosage	Schedule	Tumor weight	Tumor inhibition
				
	mg/kg	1, 5, 9 administrations	(g)	%
Control	--	--	2.36 ± 0.05	
Razoxane	80	1 time a day	2.49 ± 0.05	-5.5
Razoxane	40	1 time a day	2.32 ± 0.07	1.7
Razoxane	20	1 time a day	2.80 ± 0.06	-18.6
Razoxane	10	3 times a day*	1.51 ± 0.04**	36.0
Probimane	20	3 time a day*	1.19 ± 0.05**	49.6

**Table 5:** Antitumor effects of Raz on Lewis lung carcinoma in combination with daunorubicin: *Administration every 3 hours. Experimental term was 11 days

Compounds	Dosage	Schedule	Tumor weight (g)	Tumor inhibitions
				
	mg/kg	1, 5, and 9 administrations		%
Control			2.34 ± 0.05	
Razoxane (Raz)	10	3 times a day*	1.57 ± 0.05	32.9
Daunorubicin (Dau)	2	1 time a day	1.10 ± 0.04	53.0
Raz + Dau	10 + 2	3 times/1 time a day	1.02 ± 0.04	56.4

**Table 6:** Antitumor effects of Raz on Lewis lung carcinoma in combination with bleomycin: * Administrate every 3 hours in one day. ** p < 0.01 (treatment vs vehicle control). Experimental term was 11 days

Compounds	Dosage	Schedule	Tumor weight	Tumor Inhibition
				
	mg/kg	1, 5, and 9 administration	(g)	%
Control	--	--	2.46 ± 0.06	
Razoxane (Raz)	10	3 times a day*	1.44 ± 0.07	41.5
Bleomycin (Ble)	15	1 time a day	1.50 ± 0.06	39.0
Raz + Ble	10 + 15	3 times + 1 time a day	0.66 ± 0.05**	73.2**

**Table 7:** Antitumor effects of Pro on Lewis lung carcinoma in combination with daunorubicin or bleomycin: *Administration every 3 hours. Experimental term was 11 days

Compounds	Dosage	Schedule	Body weight	Tumor weight (g)	Tumor inhibitions
					
	mg/Kg	1, 5, and 9 administration	g		%
Control	--	--	20.6/21.6	2.62 ± 0.08	
Pro	20	3 times a day	20.6/20.8	1.45 ± 0.07	44.6
Dau	2	1 time a day	20.6/20.0	1.14 ± 0.08	56.5
Ble	15	1 time a day	20.7/21.2	1.36 ± 0.08	48.1
Pro + Dau	20 + 2	3 times/1 time a day	20.6/20.9	1.07 ± 0.05	59.2
Pro + Ble	20 + 15	3 times/1 time a day	20.7/19.8	0.59 ± 0.04	77.5

### Antitumor activity of Pro and Raz on LAX-83

The experiments showed that LAX-83 was sensitive to *Raz *(40–60 mgKg^-1^, ip × 5) and *Pro *(80–100 mgKg^-1 ^ip × 5) with inhibitory rates of 25–32 % and 55–60 % respectively (P < 0.01 vs control). *CTX*, as a positive anticancer drug (40 mgKg^-1 ^ip × 5), exhibited antitumor activities against the growth of LAX-83 with an inhibitory rate of 84 %. Obvious necrosis in tumor tissues was observed by histological evaluation of *CTX *and *Pro *treatment groups, but *Pro *showed larger vacuoles than *CTX*. Drug inhibition on tumor volumes were calculated and outlined in Table 8. We have tested the 5 most commonly used anticancer drugs – cyclophosphamide (CTX), 5-fluoruoracil (5-Fu), methotrexate (MTX), cisplatin (DDP) and vincristine (VCR) (Table 9). In the LAX-83 model, CTX has been shown to be the most effective one. The anticancer effect of *Pro *was the same or better than those of MTX, DDP and as well as 5-Fu against LAX-83 tumor growth.

**Table 8:** Antitumor activities of Pro and Raz on human tumor LAX-83 using subrenal capsule assay: Route: ip × 5 daily from the day after surgery. * P < 0.05, ** P < 0.001 (treatment vs vehicle control). Experiment was completed within 7 days. Tumor volume = 1/2 × width^2 ^× length (using T-test)

Compounds	Dosage mg/kg/d	No mice	Body weight (g)	Tumor volume (mm^3^)	Inhibition%
Control	---	16	19.2/21.0	39.8 ± 3.2	--
Razoxane	40	12	20.8/21.5	29.7 ± 3.0*	25
Razoxane	60	12	19.8/18.8	27.2 ± 2.8*	32
Probimane	80	12	20.0/19.6	18.0 ± 2.6**	55
Probimane	100	12	20.0/20.0	15.8 ± 2.6**	60
Cyclophosphamide	40	12	21.0/20.9	6.4 ± 2.0**	84

**Table 9:** Antitumor activities of anticancer drugs on human tumor LAX-83 using subrenal capsule assay: Route: ip × 5 daily from the day after surgery. * P < 0.05, ** P < 0.001 (treatment vs vehicle control). Experiment was completed within 7 days. Tumor volume = 1/2 × width^2 ^× length (using T-test)

Compounds	Dosage mg/kg/d	No mice	Body weight (g)	Tumor volume (mm^3^)	Inhibition%
Control	---	16	20.9/22.5	29.7 ± 3.2	--
Methotrexate	1.5	12	21.2/21.9	27.4 ± 3.0	7.7
Cis-platin	1.5	12	22.8/21.7	16.6 ± 2.6**	44.1
5-fluoruoracil	37.5	12	21.7/21.4	12.8 ± 2.6**	57.5
Cyclophosphamide	30.0	12	21.0/20.9	5.8 ± 2.3**	80.5
Vincristine	0.3	12	20.8/20.8	7.6 ± 2.2**	74.4

## Discussion

Explanations of anticancer and antimetastatic mechanisms of *bisdioxopiperazines *are now inconclusive. The present explanation for the anticancer mechanisms of *Raz *has been attributed to antiangiogenesis and topoisomerase II inhibition.[16] Since the antimetastatic activities of *Raz *and *Pro *were much stronger than those actions against primary tumor growth, this special targeting on metastasis ought to be more useful in clinical cancer treatment. *Raz *and *Pro *show typical characteristics of antiangiogenesis agents, which target small nodule of tumors. Meanwhile, recent reports on drugs targeting *angiogenesis *indicate that most anti-vascular drugs have low or even no effects on most cancers when they are used alone in clinics, but they show synergistic effects in combination with other anticancer drugs. [17,18] Our study shows synergistic anticancer actions of *Raz *and *Pro *with *Ble *or *Dau *basing on this theory. Previous work showed that *Pro *and *Raz *could reduce the cardiotoxicity of *anthrocycline*,[1,9,10] so we may reasonably deduce that they can also reduce the cytotoxicity of *anthrocyclines*. The data in our study suggests that the synergistic effects of *Raz *with *anthrocyclines *are present, but not as potent as those with *Ble*.

Since we have tested the antitumor activity of clinically available anticancer drugs (CTX, 5-Fu, MTX, DDP and VCR) against LAX-83, CTX being the best one, two bisdioxopiperazines studied on this work show overall similar anticancer effective as commonly used drugs. Although the anticancer effects of CTX and VCR are better than those of Pro, for other commonly used drugs, such as DDP, MTX and 5-Fu, the antitumor effects are no better than those of Pro. Since the antitumor effects of MTX and DDP are even less effective than those of *Pro *and *Raz*, we suggest that anticancer effects of *Pro *and *Raz *are within the effective anticancer ranges of commonly available anticancer drugs.

The other useful property of *Pro *is that it is the most water-soluble among the *bisdioxopiperazines*. Most *bisdioxopiperazines *are less water-soluble and given orally in clinics. Although oral administration is easy for patients, bioavailability varies from patient to patient. For some patients who have a poor absorption of *bisdioxopiperazines *in oral administration, *Pro *can be injected *iv *to maintain stable drug levels. Our previous work showed that *Pro *could strongly accumulate in tumor tissue while *Pro *levels in other tissues decrease rapidly [19]. Presently, a stereo-isomer of *Raz*, (dexrazoxane, *ICRF-187*), a water-soluble Raz, is being reinvestigated and has aroused the interests of clinical oncologists. Phase III clinical studies are currently underway in the US. More importantly, *ICRF-187 *was licensed in 28 countries in 4 continents. This work shows a noticeable inhibition of *Pro *and *Raz *on lung cancers and suggests possible usage of *Raz *and *Pro *on lung cancer in clinics.

## Conclusions

The advantages of *bisdioxopiperazines *in clinical treatment of lung cancers are as follows: (i) *Pro *and *Raz *can inhibit the growth of lung cancers, with and without the help of other anticancer drugs, like *Dau *and *Ble*; (ii) like *Raz*, *Pro *strongly inhibits spontaneous pulmonary metastasis of *LLC*; (iii) since *Pro *can inhibit *CaM *[11], a calcium activated protein that's associated with *MDR *and metastatic phenotypes, synergistic anticancer effects of *Pro *and *Raz *can be expected in combination with other anti-cancer drugs, like *Dau *or *Ble*. Now, new concepts of the relationship between tumor metastasis and *MDR *in cancers have been stated,[20] whereas *bisdioxopiperazines *can inhibit both tumor metastasis and *MDR*. As a counterpart of *Raz*, *Pro *might be of interest and have chemotherapeutic potential in clinics.

## Methods

### Drugs and animals

Cyclophosphomide (*CTX*), daunorubicin (*Dau*) and bleomycin (*Ble*), 5-fluororacil (5-Fu), vincristine (VCR), cisplatin (DDP), methotrexate (MTX) were purchased from Shanghai Pharmaceutical Company. Pro and Raz were prepared by Department of Medicinal Chemistry, Shanghai Institute of Materia Medica, Chinese Academy of Sciences. C57BL/6J and Kun-Min strain mice were purchased from Shanghai Center of Laboratory Animal Breeding, Chinese Academy of Sciences. Nude mice (Swiss-DF), taken from Roswell Park Memorial Institute, USA, were bred in Shanghai Institute of Materia Medica, Chinese Academy of Sciences under a specific pathogen free condition. Human pulmonary adenocarcinoma xenograft (*LAX-83*)[21] and Lewis lung carcinoma (*LLC*) were serially transplanted in this laboratory. All animal experiments were conducted in compliance with the Guidelines for the Care and Use of Research Animals, NIH, established by Washington University's Animal Studies Committee. Bouin's solution consists of water saturated with picric acid: formaldehyde: glacial acetic acid (75: 20: 5, v/v/v).

### Lethal dosage determination in mice

Mice of Kun-Min strain (equal amount of male and female) were *ip *injected with Pro and Raz daily for 10 successive days. The deaths of mice were counted after 1 month. Lethal dosage of agents was calculated by *Random Probity tests*.

### Antitumor and antimetastatic studies of LLC

C57BL/6J mice were implanted *sc *with *LLC *(2 × 10^6 ^cells) from donor mice. The mice were injected intraperitoneally with drugs daily or every two days for 7 injections. On day 11 or day 20, mice were sacrificed, and locally growing tumors were separated from skin and muscles and weighed, and lungs of host mice were placed into a Bouin's solution for 24 h, and then the lung samples were submerged into a solution of 95 % alcohol for 24 h. Finally, the numbers of extruding metastatic foci in lungs were counted.

### Antitumor actions of different schedules and in combinations with different drugs

C57BL/6J mice were implanted *sc *with *LLC *(2 × 10^6 ^cells) from donor mice. Mice were injected intraperitoneally with drugs on day 1, 5, 9. Single injection or 3 injections every 3 hours were used. Tumors were separated and weighed on day 11.

### Antitumor activity study of human tumors

Nude mice were inoculated with LAX-83 under the renal capsule (SRC method).[22] Nude mice were injected intraperitoneally with drugs daily during next five days after inoculation of *LAX-83*. Then nude mice were sacrificed, and their kidneys were taken out for measurement of tumor sizes using a stereomicroscope a week after transplantation. Tumor volume was calculated as 1/2(ab^2^) where a and b are their major and minor axes of the lump. Kidneys with tumors were paraffin-embedded, sliced and hematoxylin dyed. The tumor tissues were then observed from a light microscope.

### Statistical analysis

*Student's t-test *was used to assess the differences between control and drug treatment groups of above methods.

## List of abbreviation used are

Pro, probimane; Raz, razoxane; CaM, calmodulin; LPO, lipoperoxidation; Dau, daunorubicin; Ble, bleomycin; LLC, Lewis lung carcinoma, LAX-83; a lung adenocarcinoma xenograft; ADR, adriamycin;

## Author's contribution

The experimental design was made by Bin Xu and Da-Yong Lu.

Experiments were performed by Da-Yong Lu (anticancer activity tests)

The manuscript was written by Da-Yong Lu, and Jian Ding.

**Figure 1:**
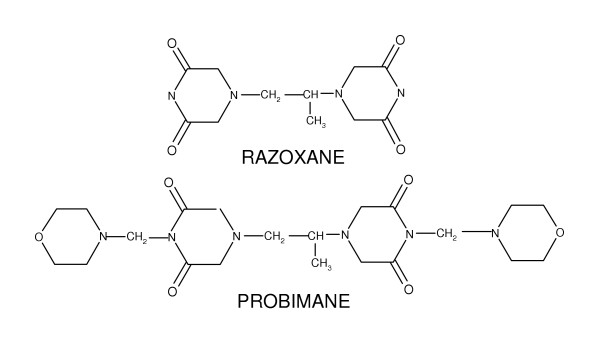
Structural formulas of razoxane and probimane

## References

[B1] Herman EH, Witiak DT, Hellmann K, Waradek VS (1982). Properties of ICRF-159 and related Bis(dioxopiperazine) compounds. Advances in Pharmacology and Chemotherapy.

[B2] Hellmann K, Rhomberg W (1991). Radiotherapeutic enhancement by razoxane. Cancer Treat Rev.

[B3] Sargent JM, Williamson CJ, Yardley C, Taylor CG, Hellmann K (2001). Dexrazoxane significantly impares the induction of doxorubicin resistance the human leukemia line, K562. Brit J Cancer.

[B4] van Hille B, Etievant C, Barret JM, Kruczynski A, Hill BT (2000). Characterization of the biological and biochemical activities of F 11782 and bisdioxopiperazines, ICRF-187 and ICRF-193, two types of topoisomerase II catalytic inhibitors with distinctive mechanisms of action. Anti-cancer Drugs.

[B5] Ji RY (1988). Probimane. Drugs Fut.

[B6] Wang MY, Liu TX, Li GT, Zhang TM (1988). Effects of bimolane and probimane on the incorporation of [^3^H]TdR, [^3^H]UR and [^3^H]Leu into Ehrlich ascites carcinoma cells *in vitro*. Zhongguo Yuo Li Xue Bao.

[B7] Zhang TM, Wang MY, Wang QD (1987). Antineoplastic action and toxicity of probimane and its effect on immunologic functions in mice. Zhongguo Yao Li Xue Bao.

[B8] Yang KZ, Huang BY, Huang TH, Wu YD (1990). Short-term results of malignant lymphoma treated with probimane. Chin J Cancer.

[B9] Zhang Y, Hua HY, Zhang TM (1993). Inhibitory effect of dioxopiperazine compounds on malondialdehyde formation induced by doxorubicin in rat liver mitochondria *in vitro*. Zhongguo Yao Li Xue Bao.

[B10] Zhang Y, Liu J, Wang J, Ye QX, Zhang TM (1997). Effects of probimane (Pro) and doxorubicin (Dox) in combination on DNA synthesis and cell cycle of tumor cells. Chin Pharmacol Bull.

[B11] Lu DY, Chen EH, Cao JY, Zhou JJ, Shen ZM, Xu B, Horie K (2001). Comparison between probimane and razoxane on inhibiting calmodulin activity of rabbit erythrocyte membrane. Chin J Pharmacol Toxicol.

[B12] Lu DY, Chen EH, Cao JY, Jin W, Tian F, Ding J (2003). The inhibition of probimane on lipid peroxidation of rabbit and human erythrocytes. J Shanghai Univ (Eng).

[B13] Lu DY, Liang G, Zhang MJ, Xu B (1994). Serum contents of sialic acids in mice bearing different tumors. Chin Sci Bull (Eng).

[B14] Lu DY, Chi J, Lin LP, Huang M, Xu B, Ding J (2004). Effects of anticancer drugs on the binding of ^125^I-fibrinogen to two leukemia cells *in vitro*. J Int Med Res.

[B15] Nishio K, Nakamura T, Koh Y, Suzuki H, Fukumoto N, Saijo N (1999). Drug resistance in lung cancer. Current Opin Oncol.

[B16] Braybrooke JP, O'Byrne KJ, Propper DJ, Blann A, Saunders M, Dobbs N, Han C, Woodhull J, Mitchell K, Crew J, Smith K, Stephens R, Ganesan T, Talbot DC, Harris AL (2000). A phase II study of razoxane, an antiangiogenic topoisomerase II inhibitor, in renal cell cancer with assessment of potential surrogate markers of angiogenesis. Clin Cancer Res.

[B17] Taraboletti G, Margosio B (2001). Antiangiogenic and antivascular therapy for cancer. Current Opinion in Pharmacology.

[B18] Mark J (2003). A boost for tumor starvation. Science (Washington DC).

[B19] Yang JM, Xu ZD, Wu H, Zhu HG, Wu XH, Hait WN (2003). Overexpression of extracellular matrix metalloproteinase inducer in multidrug resistant cancer cells. Molecular Cancer Res.

[B20] Lu DY, Xu B, Zhang X, Chen RT (1993). Distribution of ^14^C labeled at dioxopiperazine or methyl morphorline group of probimane by whole body autoradiography. Zhongguo Yuo Li Xue Bao.

[B21] Zhang SY, Dai ZQ, Shu YH, Zhang JL, Lin ZQ, Wu SF, Liu YF, Xu LZ (1987). Establishment of human lung adenocarcinoma model in nude mice and sensitivity of the transplanted tumor to antitumor drugs. Zhongguo Yuo Li Xue Bao.

[B22] Wang XY, Ling CQ (2002). Tumor chemosensitivity test *in vivo *and subrenal capsule assay. Chin J Cancer.

